# A fault-tolerant addressable spin qubit in a natural silicon quantum dot

**DOI:** 10.1126/sciadv.1600694

**Published:** 2016-08-12

**Authors:** Kenta Takeda, Jun Kamioka, Tomohiro Otsuka, Jun Yoneda, Takashi Nakajima, Matthieu R. Delbecq, Shinichi Amaha, Giles Allison, Tetsuo Kodera, Shunri Oda, Seigo Tarucha

**Affiliations:** 1 RIKEN, Center for Emergent Matter Science, Wako-shi, Saitama 351-0198, Japan.; 2 Department of Physical Electronics and Quantum Nanoelectronics Research Center, Tokyo Institute of Technology, O-okayama, Meguro-ku, Tokyo 152-8552, Japan.; 3 Department of Applied Physics, University of Tokyo, Hongo, Bunkyo-ku, Tokyo 113-8656, Japan.

**Keywords:** physics, Nanotechnology, spin qubit, qubit, quantum dot, Si/SiGe, Silicon

## Abstract

Fault-tolerant quantum computing requires high-fidelity qubits. This has been achieved in various solid-state systems, including isotopically purified silicon, but is yet to be accomplished in industry-standard natural (unpurified) silicon, mainly as a result of the dephasing caused by residual nuclear spins. This high fidelity can be achieved by speeding up the qubit operation and/or prolonging the dephasing time, that is, increasing the Rabi oscillation quality factor *Q* (the Rabi oscillation decay time divided by the π rotation time). In isotopically purified silicon quantum dots, only the second approach has been used, leaving the qubit operation slow. We apply the first approach to demonstrate an addressable fault-tolerant qubit using a natural silicon double quantum dot with a micromagnet that is optimally designed for fast spin control. This optimized design allows access to Rabi frequencies up to 35 MHz, which is two orders of magnitude greater than that achieved in previous studies. We find the optimum *Q* = 140 in such high-frequency range at a Rabi frequency of 10 MHz. This leads to a qubit fidelity of 99.6% measured via randomized benchmarking, which is the highest reported for natural silicon qubits and comparable to that obtained in isotopically purified silicon quantum dot–based qubits. This result can inspire contributions to quantum computing from industrial communities.

## Teaser

This is the first experimental demonstration of a fault-tolerant spin qubit in industry-compatible isotopically natural silicon.

## INTRODUCTION

Since the proposal of spin qubits using electrons confined in quantum dots (*1*), a great deal of effort has been made to implement quantum dot–based spin qubits in a variety of semiconductors, such as group III-V compounds (*2*–*7*) or natural silicon (*8*–*10*). However, the quantum gate fidelities in these qubits are limited, mainly because of the short coherence time [$T_{2}^{*}$ < 0.1 μs (*2*, *3*, *6*, *7*) for group III-V compounds and $T_{2}^{*}$ < 1 μs for natural silicon (*8*–*10*)] caused by the nuclear spin magnetic field fluctuations. A straightforward approach to obtain a qubit fidelity higher than the quantum error correction threshold for fault-tolerant quantum computing (*11*, *12*) is to prolong the qubit decay time $T_{2}^{\text{Rabi}}$ or shorten the π rotation time *T*_π_ to increase the qubit Rabi oscillation quality factor $Q=T_{2}^{\text{Rabi}}/T_{\pi }$ because it determines the upper bound of the qubit fidelity.

The first approach has been implemented in an isotopically purified silicon qubit with a long coherence time, while leaving the spin control time [*T*_π_ = 1.6 μs (*13*)] much slower than the other quantum dot–based spin qubits [*T*_π_ = 0.005 μs (*7*, *14*)]. For the realization of fault-tolerant qubits in more common materials, such as natural silicon, one important issue that must be resolved is the slow spin control time (*13*, *15*). The key parameter to realize the fast spin control is a large (effective) oscillating magnetic field to drive the spin resonance, while keeping the applied microwave power small enough to suppress unwanted effects, such as photon-assisted tunneling (*3*, *7*) or heating. However, because silicon does not have any strong spin driving mechanisms, such as spin-orbit interaction (*7*, *16*), an on-chip coplanar stripline is commonly used as a method to generate an oscillating magnetic field (*13*, *15*) despite being unsuitable for generating a large magnetic field. Alternatively, a micromagnet technique (*17*) can be used to implement a material-independent artificial strong spin-orbit coupling (*14*), resulting in a much stronger effective magnetic field. Therefore, the micromagnet technique may help to increase the quality factor for natural silicon quantum dot qubits comparable to that in isotopically purified silicon but in a much higher Rabi frequency range. Recently, the technique has been applied to a silicon single quantum dot (*10*), and an improvement of the Rabi frequency (*f*_Rabi_) by an order of magnitude was achieved, although the device structure was not appropriately optimized for fast and addressable control.

## RESULTS

Here, we report an addressable fault-tolerant qubit using a natural silicon double quantum dot with a micromagnet that is optimally designed for fast spin control. From microwave spectroscopy, a resonance frequency difference of about 800 MHz is obtained for the two electron spins confined in each quantum dot. This result shows the good addressability of our qubits because the obtained frequency difference is about two orders of magnitude larger than our fast Rabi frequency and the crosstalk error is as small as 0.02% for our typical Rabi frequency of 10 MHz. The qubit dephasing time $T_{2}^{*}$ is measured by Ramsey interference. It shows a standard Gaussian decay with a $T_{2}^{*}$ of about 2 μs caused by the nuclear spin fluctuations. The two-axis single-qubit control is confirmed by the observation of the shift of Ramsey fringe by modulating the relative phase of the second microwave burst. To operate the qubit much faster than its decay rate or to maximize the Rabi oscillation quality factor, the microwave amplitude (*A*_MW_) dependence of *f*_Rabi_ and $T_{2}^{\text{Rabi}}$ is measured. The optimized *A*_MW_ corresponds to *f*_Rabi_ ~ 10 MHz, which is about two orders of magnitude faster than the reported value for an isotopically purified silicon quantum dot (*13*), owing to the effectiveness of the magnetic field generation by our optimized micromagnet. Finally, Clifford-based randomized benchmarking is performed to determine the qubit fidelity. At the optimized *A*_MW_, an average single-qubit fidelity of 99.6% is obtained. The qubit fidelity is reduced for the condition with shorter *T*_π_ and smaller *Q*, which indicates that it is limited by heating at large *A*_MW_.

**Fig. 1:**
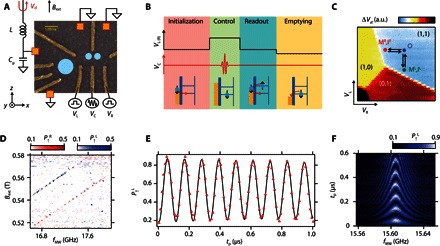
Device structure and EDSR measurement result. (**A**) False-color scanning electron micrograph of the device. The orange boxes represent ohmic contacts that are grounded during the measurements except for the one connected to the resonance circuit. The two small circles show the approximate position of the double quantum dot, and the large circle shows the approximate position of the sensor quantum dot. Three of the gate electrodes (R, L, and C) are connected to impedance-matched high-frequency lines with cryogenic bias tees. (**B**) Schematic of the pulse sequence used for the EDSR measurement. The pulse sequence consists of four stages, namely, initialization, control, readout, and emptying. (**C**) Charge stability diagram in the vicinity of the (1,1) charge configuration. M^R^,I^R^ (M^L^,I^L^) denote the measurement and initialization points for the right (left) quantum dot. O denotes the operation point that is common for both right and left quantum dots. a.u., arbitrary units. (**D**) Measurement of the EDSR signal as a function of *f*_MW_ and *B*_ext_. The blue line corresponds to the left dot resonance condition *hf*_MW_ = *g*μ(*B*_ext_ + $B_{z}^{\text{MM},L}$), and the red line corresponds to the right dot resonance condition *hf*_MW_ = *g*μ(*B*_ext_ + $B_{z}^{\text{MM},R}$). (**E**) Rabi oscillation with $T_{2}^{\text{Rabi}}$ ~ 8 μs and *f*_Rabi_ ~ 9 MHz measured at *B*_ext_ = 0.505 T and *f*_MW_ = 15.6055 GHz. The red triangles show measurement data, and the black solid line shows the fitting with an exponentially damped sine curve, $P_{↑}(t_{p})=A\;\text{exp}(-t_{p}/T_{2}^{\text{Rabi}})\;\text{sin}(2\pi f_{\text{Rabi}}t_{p}+\theta )+B$ with *A*, *B*, *f*_Rabi_, θ, and $T_{2}^{\text{Rabi}}$ as fitting parameters. (**F**) Measurement result of detuned Rabi oscillations, which shows a typical chevron pattern.

Our double quantum dot in silicon is formed by locally depleting a two-dimensional electron gas in an undoped natural Si/SiGe heterostructure by lithographically defined electrostatic gates (Fig. 1A). A 250-nm-thick cobalt micromagnet is placed on top of the device to induce a stray magnetic field around the quantum dot. To achieve the fast and addressable control of single-electron spins using electric dipole spin resonance (EDSR), the micromagnet is designed to maximize the slanting magnetic field, $dB_{y}^{\text{MM}}/\text{dz}$, and the local Zeeman field difference between the two dots (*14*), $\Delta B_{z}=|B_{z}^{\text{MM},R}-B_{z}^{\text{MM},L}|$, where *B*^MM, R(L)^ denotes the stray magnetic field at the right (left) dot position. This micromagnet enables a slanting field several times larger than that obtained by Kawakami *et al*. (*10*) (the micromagnet simulation is provided in section S1). A nearby sensor quantum dot coupled to a radio-frequency tank circuit allows rapid measurement of the double quantum dot charge configuration (*18*). The sample is cooled down to a base electron temperature of 120 mK estimated from the linewidth of the dot transport, using a dilution refrigerator. An in-plane external magnetic field *B*_ext_ is applied using a superconducting magnet. The double quantum dot is tuned to the (1,1) charge state where each dot hosts only one electron (Fig. 1C). Single-shot measurement of the spin state is performed using an energy-selective readout technique (fig. S2) (*19*).

Figure 1B shows the pulse sequence for the spin control. First, the spin-down state is initialized by applying gate voltages such that only the ground spin-down state can tunnel into the dot. Next, the gate voltages are pulsed so that the electrons confined in the dot are pushed deep in Coulomb blockade. Then, a microwave burst with a frequency of *f*_MW_ is applied to gate C to induce EDSR. Finally, the gate voltages are pulsed back to the spin readout position where only a spin-up electron can tunnel out to the reservoir. An additional emptying (or compensation) stage is used to keep the dc offset of the pulse to zero. When the microwave burst is applied to the gate, the wave function of electrons confined in the dot oscillates spatially in the slanting magnetic field induced by the micromagnet, resulting in an effective oscillating magnetic field *B*_AC_ perpendicular to the static magnetic field $B_{0}=B_{\text{ext}}+B_{z}^{\text{MM}}$. Under the condition where *f*_MW_ = *g*μ*B*_0_ (*g* is the electron *g*-factor and μ is the Bohr magnetron), EDSR takes place. The resonance conditions are different for each dot by an amount proportional to ΔB_z_, and therefore, the resonances of each dot can be addressed independently.

Figure 1D shows the spin-up probabilities for both right ($P_{↑}^{R}$, red signal) and left ($P_{↑}^{L}$, blue signal) dot as a function of *B*_ext_ and *f*_MW_. For this measurement, a rectangular microwave burst with a fixed duration of *t*_p_ = 3 μs is applied. There are two clear resonance lines corresponding to each dot, which are separated by ΔB_z_ ~ 30 mT or 800 MHz, consistent with our micromagnet simulation (fig. S1C). The observed 800-MHz splitting is approximately two orders of magnitude larger than the value obtained for the spin-orbit–mediated Stark shift of g*-factor in a silicon quantum dot spin qubit without a micromagnet (*13*). For multiple qubit systems, it is crucial to have such a large frequency splitting to operate the qubit independently without crosstalk because the effect of the driving field decays with (*f*_Rabi_)^2^/((Δ*f*)^2^ + (*f*_Rabi_)^2^), where $\Delta f=f_{\text{res}}^{R(L)}-f_{\text{MW}}$ is the frequency detuning from the center resonance frequency. For our typical *f*_Rabi_ of 10 MHz, the 800-MHz splitting yields a crosstalk operation of the idle qubit with an amplitude as small as 0.02% of the operated qubit. For the following measurements, we mainly focus on the left quantum dot, as the sensitivity of our charge sensor is significantly higher owing to the design of our device. However, the right quantum dot shows similar results, as detailed in section S3.

The coherent evolution of the spin state is measured by changing the microwave duration at the resonance frequency (Fig. 1E). The red triangles show the experimental data, and the black solid line shows a fit with an exponentially damped sinusoidal function with a Rabi oscillation decay time $T_{2}^{\text{Rabi}}$ of 8 μs and an *f*_Rabi_ of 9 MHz (details on the fitting procedure are provided in section S4). Note that the $T_{2}^{\text{Rabi}}$ of a strongly driven qubit is different from the standard $T_{2}^{*}$ dephasing time measured from Ramsey interference because the influence of nuclear spin fluctuations is suppressed by the Rabi driving field (*14*). When *f*_MW_ is detuned from the resonance frequency, the qubit rotates around a tilted axis in the Bloch sphere. This results in faster rotation at detuned *f*_MW_ and the chevron pattern shown in Fig. 1F.

**Fig. 2:**
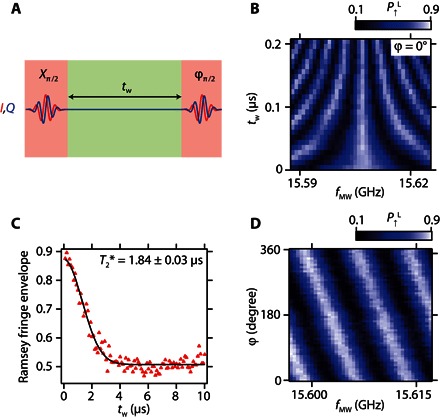
Ramsey interference measurements. (**A**) Schematic of the Ramsey measurement sequence. ϕ denotes the phase of the second microwave burst relative to the first X_π/2_ rotation. A rectangle or Gaussian microwave burst is applied to gate C. (**B**) Ramsey fringes measurement result. *B*_ext_ is fixed at 0.505 T. ϕ is the phase of the second microwave burst relative to the first microwave burst. (**C**) Ramsey fringes decay envelope extracted by sweeping *f*_MW_ at each fixed *t*_w_. The black solid line is a fit with a Gaussian decay function $P_{↑}^{L}(t_{w})=A\;\text{exp}(-{\left(t_{w}/T_{2}^{*}\right)}^{2})+B$, where *A* and *B* are constants to account for the measurement and initialization fidelities. (**D**) Demonstration of the π/2 pulse around an arbitrary rotation axis in the *xy* plane of the Bloch sphere.

**Fig. 3:**
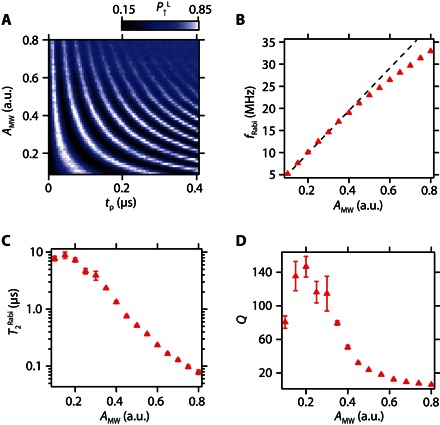
Rabi oscillation power dependence. (**A**) Microwave amplitude dependence of Rabi oscillations measured at *B*_ext_ = 0.505 T and *f*_MW_ = 15.6055 GHz. (**B**) Microwave amplitude dependence of the Rabi frequency *f*_Rabi_. The red triangles show the measured data, and the black dotted line shows a linear fitting for the small-amplitude data (0.1 ≤ *A*_MW_ ≤ 0.25). The fitting error is smaller than the size of the symbols. (**C**) Microwave amplitude dependence of the Rabi decay time $T_{2}^{\text{Rabi}}$. Because the total evolution time of the data used for the fitting is relatively short (*t*_p_ = 3 μs), it shows large errors for small *A*_MW_ points. (**D**) Microwave amplitude dependence of the quality factor $Q=T_{2}^{\text{Rabi}}/T_{\pi }$. The error mainly comes from the uncertainty of $T_{2}^{\text{Rabi}}$.

Next, characterization of the qubit coherence time is performed using a Ramsey interference technique (Fig. 2A). First, the spin is initialized in the down state. Then, a π/2 pulse is applied to rotate the spin to the equator of the Bloch sphere where it accumulates a phase error for the wait time *t*_w_. Finally, the spin state is rotated by the second π/2 pulse to project the phase error to the *z* axis. Figure 2B presents a Ramsey measurement result that shows well-defined Ramsey fringes. From the decrease of the Ramsey fringe amplitude as a function of *t*_w_, the value of the dephasing time $T_{2}^{*}$ can be obtained (Fig. 2C). The red triangles show measured data, and the black solid line is a fit with a Gaussian decay function with a $T_{2}^{*}$ of 1.83 μs. The Gaussian decay indicates that the system noise is predominantly white because of the nuclear spin noise and/or the charge noise. Note that the charge noise can be a magnetic noise source in the presence of the field gradient (*20*, *21*). Because the measured $T_{2}^{*}$ is the longest value observed in quantum dot spin qubit systems based on isotopically natural silicon (*8*–*10*), we speculate that the charge noise does not significantly limit $T_{2}^{*}$ in our experiment. Two-axis control in the Bloch sphere is required for arbitrary spin qubit control. This is demonstrated by modulating the phase φ of the second microwave burst in the Ramsey measurement. The observed shift of the Ramsey fringe in Fig. 2D corresponds to the change of rotation angle of the second π/2 rotation. To improve the qubit fidelity by suppressing the influence of dephasing, it is straightforward to maximize the Rabi oscillation quality factor $Q=T_{2}^{\text{Rabi}}/T_{\pi }$ by applying a larger microwave excitation to decrease the π rotation time *T*_π_. However, it has been reported that a very large microwave excitation can cause significant dephasing due to photon-assisted tunneling (*4*, *14*, *22*); therefore, it is important to characterize the microwave amplitude (*A*_MW_) dependence of the Rabi oscillation. *A*_MW_ is in units directly proportional to the actual voltage. As *A*_MW_ is increased, the oscillation period becomes shorter; however, the signal is damped more rapidly (Fig. 3A). By fitting the data of the damped oscillations with exponentially decaying functions (see section S4 for the details), *f*_Rabi_ and $T_{2}^{\text{Rabi}}$ are extracted as a function of *A*_MW_ (Fig. 3B). *f*_Rabi_ increases with *A*_MW_ linearly when *A*_MW_ is smaller than about 0.3 but finally shows a deviation from the linear relation (*14*). The deviation from the linear relation is probably due to the anharmonicity of the quantum dot confinement potential (*17*). The obtained maximum *f*_Rabi_ is about 35 MHz, showing an improvement of one or two orders of magnitude from previous experiments (*10*, *13*). $T_{2}^{\text{Rabi}}$ shows a significant decrease when *A*_MW_ becomes larger. Here, the photon-assisted tunneling mechanism may be ruled out because the decrease of $T_{2}^{\text{Rabi}}$ does not strongly depend on the depth of Coulomb blockade or the operation point (fig. S5). Alternatively, heating due to the microwave burst, which causes the reduction of *T*_2_ rather than $T_{2}^{*}$, can be a dominant source of the observed decay as previously observed in a singlet-triplet qubit (*23*). Figure 3D shows the quality factor *Q* of the Rabi oscillations as a function of *A*_MW_. From these data, the optimal working point for the qubit operation is estimated to be *A*_MW_ ~ 0.2, where *f*_Rabi_ ~ 10 MHz and *Q* ~ 140 are obtained. The obtained maximum *Q* is in the same range as the one in an isotopically purified silicon quantum dot with two orders of magnitude slower *f*_Rabi_ and two orders of magnitude longer $T_{2}^{\text{Rabi}}$ (*13*).

**Fig. 4:**
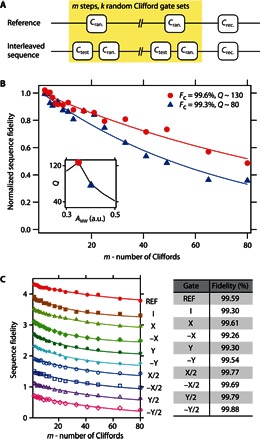
Randomized benchmarking measurement. (**A**) Schematic of the randomized benchmarking sequence. The upper panel is a reference sequence consisting of *m* random Clifford gates. The lower panel is the interleaved sequence used to measure fidelities of a specific test Clifford gate *C*_test_. The sequence is repeated for *k* = 16 choices of sequences to obtain one point. (**B**) Reference randomized benchmarking for two different microwave amplitudes. The inset shows the quality factor measurement for Gaussian microwave burst, which shows a result that is similar to the one for rectangle microwave burst. (**C**) Interleaved randomized benchmarking for single-step Clifford gates. The table on the right shows fidelity measurement results for several single-qubit gates. The fitting error of each gate fidelity is smaller than 0.1% for the reference and all interleaving measurements.

Finally, the single-qubit control fidelity is characterized via randomized benchmarking (*24*) using the measurement sequence shown in Fig. 4A. The reference sequence includes *m* random Clifford gates and one recovery Clifford gate chosen such that the ideal final spin state becomes an eigenstate of σ_z_. The interleaving sequence (*25*) is used to determine the fidelity of each single-step Clifford gate *C*_test_. The pulse envelope is shaped to a Gaussian (truncated at ± 2σ) to minimize its spectral width for suppressing pulse errors (*26*). An interval of 6 ns between gate operations is used to avoid pulse overlap. Figure 4B shows the reference randomized benchmarking measurement to determine the averaged Clifford gate fidelity. By fitting the reference measurement with an exponentially decaying curve, *F*(*m*) = *A*(2*F*_c_ − 1)^*m*^, where *A* is the visibility and *F*_c_ is the Clifford gate fidelity per step, which corresponds to 1.875 single Clifford gates (details are provided in section S5). At the optimized *A*_MW_, to maximize *Q* (the red point in the inset of Fig. 4B), we obtain $F_{c}^{\text{single}}$ = 99.6%, which is above the threshold for fault-tolerant quantum computing (*12*) and comparable to the value reported in an isotopically purified silicon quantum dot (*13*). Note that $F_{c}^{\text{single}}$ is decreased for a larger *A*_MW_ or shorter *T*_π_ (the blue point in the inset of Fig. 4B), indicating that *Q* is a good indicator to optimize *A*_MW_ and $F_{c}^{\text{single}}$. Figure 4C shows interleaving measurements for the single-step Gaussian Clifford gates. As expected from the microwave heating, it is found that the fidelities for π/2 gates are higher than those for π gates. This result is consistent with our qubit decay time–limiting mechanism, the microwave effect.

## DISCUSSION

The Si/SiGe double quantum dot used in this work contains an optimized micromagnet that enables Rabi frequencies that are nearly two orders of magnitude faster than the values reported in previous studies without causing too much decoherence by unwanted photon-assisted tunneling or heating effects at high microwave amplitudes. From the microwave amplitude dependence measurement, we find the optimum working point to maximize the Rabi oscillation quality factor *Q*. The maximum *Q* of 140 is obtained at *f*_Rabi_ = 10 MHz, which is much faster than the qubit decay rate (1/$T_{2}^{\text{Rabi}}$ ~ 140 kHz). Together with the large qubit resonance frequency difference due to the large micromagnet inhomogeneous field (resonance frequency difference of ~800 MHz), we can implement fault-tolerant, fast, and addressable single-spin qubit operations even without the use of rare isotopically purified silicon. This may facilitate the realization of a large-scale quantum processor using existing industry-standard silicon nanofabrication techniques. Our micromagnet technique can also be applied to isotopically purified silicon to further enhance the Rabi oscillation quality factor *Q* and the qubit fidelity. We also note that it is possible to realize further enhancements by several trivial optimizations with natural silicon devices [for example, decreasing the distance between the dot and the micromagnet (*14*) or changing the gate geometry to increase the microwave gate lever arm] to increase the effective ac magnetic field strength and *f*_Rabi_. Although the effect of charge noise–induced dephasing is not clearly observed in this work, it is important to balance the effect of the Rabi frequency enhancement and the dephasing caused by the charge noise to maximize *Q* and the resulting qubit fidelity. The two-qubit gate for this system can be implemented in a straightforward manner using the exchange interaction (*15*, *27*). Because the performance of the two-qubit gate appears to be limited by charge noise, the use of natural silicon may not limit the two-qubit operation.

## MATERIALS AND METHODS

The undoped Si/SiGe heterostructure used in this study was grown by chemical vapor deposition. The surface of the heterostructure was covered by a 10-nm-thick Al_2_O_3_ insulator formed by atomic layer deposition. The ohmic contacts were fabricated by phosphorus ion implantation. The quantum dot confinement gates and the accumulation gate were formed by electron beam lithography and metal deposition. The accumulation gate and depletion gate electrodes were separated from each other by another 50-nm-thick Al_2_O_3_ insulator layer. A 250-nm-thick cobalt micromagnet was deposited on top of the accumulation gate to induce a stray magnetic field around the quantum dot. The distance between the micromagnet and the Si quantum well was 162 nm.

The sample was cooled down using a dilution refrigerator to a base electron temperature of 120 mK, which was estimated from the transport linewidth. The gates R, L, and C were connected to high-frequency coaxial lines for application of the gate voltage pulse and the microwave burst. Rapid measurement of the charge state was performed by radio-frequency reflectometry of a sensor quantum dot. More detailed information about the device structure and measurement setup are available in the Supplementary Materials.

## Supplementary Material

http://advances.sciencemag.org/cgi/content/full/2/8/e1600694/DC1

## SUPPLEMENTARY MATERIALS

Supplementary material for this article is available at http://advances.sciencemag.org/cgi/content/full/2/8/e1600694/DC1 section S1. Sample structure and micromagnet simulation section S2. Measurement setup section S3. Right dot measurement data section S4. Discussions on the microwave power dependence section S5. Randomized benchmarking fig. S1. Micromagnet design and simulation. fig. S2. Single-shot spin readout using energy selective readout technique. fig. S3. Rabi oscillation and Ramsey measurements of the right quantum dot. fig. S4. Fitting of Rabi oscillation data. fig. S5. Rabi decay measurement for two different operation points. References (*28*–*31*)
