# Supplementary material for: A fault-tolerant addressable spin qubit in a natural silicon quantum dot
Source: Sci Adv. 2016 Aug 12;2(8):e1600694. doi: 10.1126/sciadv.1600694 (PMC4982751; doi:10.1126/sciadv.1600694)
Supplement: http://advances.sciencemag.org/cgi/content/full/2/8/e1600694/DC1 [file supp_2_8_e1600694__index.html]

Science Advances | Science Advances

## Supplementary Materials

**This PDF file includes:**

- section S1. Sample structure and micromagnet simulation
- section S2. Measurement setup
- section S3. Right dot measurement data
- section S4. Discussions on the microwave power dependence
- section S5. Randomized benchmarking
- fig. S1. Micromagnet design and simulation.
- fig. S2. Single-shot spin readout using energy selective readout technique.
- fig. S3. Rabi oscillation and Ramsey measurements of the right quantum dot.
- fig. S4. Fitting of Rabi oscillation data.
- fig. S5. Rabi decay measurement for two different operation points.
- References (*28*–*31*)

Download PDF

**Files in this Data Supplement:**

- Adobe PDF - 1600694\_SM.pdf
