# Supplementary material for: A fault-tolerant addressable spin qubit in a natural silicon quantum dot
Source: Sci Adv. 2016 Aug 12;2(8):e1600694. doi: 10.1126/sciadv.1600694 (PMC4982751; doi:10.1126/sciadv.1600694)
Supplement: http://advances.sciencemag.org/cgi/content/full/2/8/e1600694/DC1 [file 1600694_SM.pdf]

Science Advances

AAAS

advances.sciencemag.org/cgi/content/full/2/8/e1600694/DC1

Supplementary Materials for

A fault-tolerant addressable spin qubit in a natural silicon quantum dot

Kenta Takeda, Jun Kamioka, Tomohiro Otsuka, Jun Yoneda, Takashi Nakajima, Matthieu R. Delbecq, Shinichi Amaha, Giles Allison, Tetsuo Kodera, Shunri Oda, Seigo Tarucha

Published 12 August 2016, *Sci. Adv.* **2**, e1600694 (2016)
DOI: 10.1126/sciadv.1600694

This PDF file includes:

- section S1. Sample structure and micromagnet simulation
- section S2. Measurement setup
- section S3. Right dot measurement data
- section S4. Discussions on the microwave power dependence
- section S5. Randomized benchmarking
- fig. S1. Micromagnet design and simulation.
- fig. S2. Single-shot spin readout using energy selective readout technique.
- fig. S3. Rabi oscillation and Ramsey measurements of the right quantum dot.
- fig. S4. Fitting of Rabi oscillation data.
- fig. S5. Rabi decay measurement for two different operation points.
- References (*28*–*31*)

section S1. Sample structure and micromagnet simulation

The layer sequence of the device is shown in fig. S1A. The undoped Si/SiGe heterostructure used in this study is grown by chemical vapor deposition. First a 3 μm thick graded buffer is grown on a Si substrate by linearly increasing the Ge content from 0 % to 30 %, and then a 1 μm thick $Si_{0.7}Ge_{0.3}$ buffer is grown. Next, a 15 nm thick Si quantum well, a 60 nm thick undoped $Si_{0.7}Ge_{0.3}$ spacer, and a 2 nm thick Si cap are successively grown on the buffer. The resulting two-dimensional electron gas has a typical electron density of 5.0×10^{11} cm^{-2} and a mobility of 1.8×10^{5} cm^{2}/Vs at an accumulation gate voltage of 1.5 V and a bath temperature of T=2.3 K.

The surface of the heterostructure is covered by a 10 nm thick Al_2O_3 insulator formed by atomic layer deposition. The ohmic contacts are fabricated by phosphorus ion implantation.

The quantum dot confinement gates and the accumulation gate are formed by electron-beam lithography and metal deposition. The accumulation gate and depletion gate electrodes are separated from each other by another 50 nm thick Al_2O_3 insulator layer.

A 250 nm thick cobalt micromagnet is deposited on top of the accumulation gate to induce a stray magnetic field around the quantum dot. The distance between the micromagnet and the Si quantum well is 162 nm.

The micromagnet design is adopted from our previous GaAs double quantum dot device (14) (fig. S1B). The design is optimized to obtain the large slanting field and the local Zeeman field difference to maximize spin rotation speed and to minimize the single-qubit operation crosstalk errors. The micromagnet magnetic field simulation is performed by using the method as in Refs. 14, 28 (We use a Mathematica package Radia, http://www.esrf.eu/Accelerators/Groups/InsertionDevices/Software/Radia). According to the calculation, the slanting magnetic field is $\mathrm{d}B_{\mathrm{y}}^{\mathrm{MM,R}}/\mathrm{dz}$ =0.8 T/μm for the right quantum dot and $\mathrm{d}B_{\mathrm{y}}^{\mathrm{MM,L}}/\mathrm{dz}$ =0.75 T/μm for the left quantum dot and the local Zeeman field difference is $\Delta B_{\mathrm{z}}=\left|B_{\mathrm{z}}^{\mathrm{MM,R}}-B_{\mathrm{z}}^{\mathrm{MM,L}}\right|$= 29 mT. The local Zeeman field difference is roughly consistent with our observation (see Fig. 1D). On the other hand, estimating the slanting field from the experimental data is difficult, since f_{Rabi} is affected by many parameters such as the microwave gate lever arm for the dot position, the electric field direction at the dot position etc., which are difficult to measure experimentally.

The valley splitting is confirmed to be larger than the Zeeman splitting by

magneto-spectroscopy measurement. Therefore the physics in this work is mainly described by a conventional single-valley picture, although there may be a small fraction of the population in the excited valley state due to the initialization error.

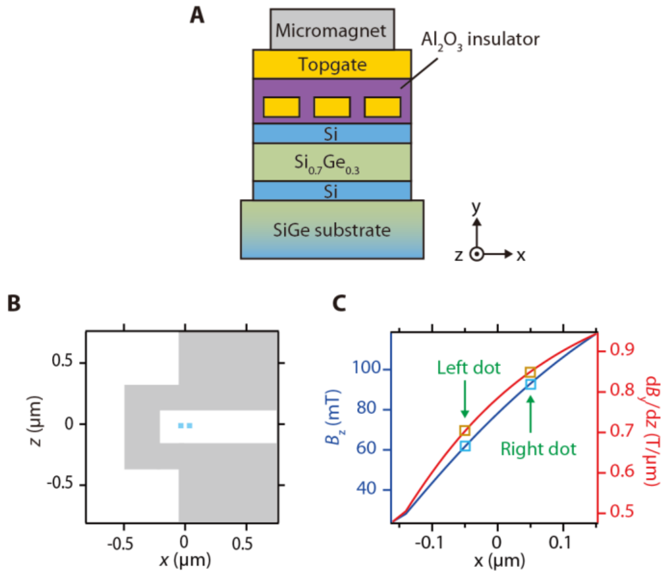

fig. S1. Micromagnet design and simulation. (**A**) Schematic layer sequence of the device structure. The external magnetic field is applied along the positive z-direction. (**B**) Schematic of the micromagnet design. The gray area shows the micromagnet pattern and the quantum dot locations are represented by the blue boxes separated by 0.1 μm. (**C**) Simulated slanting magnetic field dB_y^{MM}/dz (red curve) and local Zeeman field B_z^{MM} (blue curve) as a function of the lateral dot position *x*. The left dot position is *x*=-0.05 μm and the right dot position is *x*=0.05 μm.

section S2. Measurement setup

The sample is cooled down using a dilution refrigerator to a base electron temperature of 120 mK which is estimated from the transport linewidth. The gates R, L and C are connected to high-frequency coaxial lines for application of the gate voltage pulse and the microwave burst.

The high-frequency lines are attenuated inside the dilution refrigerator to dissipate the Johnson-Nyquist and technical noises from the room-temperature electronics (-33 dB for gates R and L, -13 dB for gate C). The voltage pulse to the gate electrodes is generated by a Sony/TektronixAWG520 arbitrary waveform generator (typical jitter 18 ps),

and applied via cryogenic bias-tees with a cut-off frequency of ~ 50 Hz. The microwave signal is applied to gate C using an Agilent E8267D vector microwave signal generator (amplitude stability 0.01dB/deg C). The output power is fixed at +16 dBm except for the randomized benchmarking measurement which requires fine tuning of the microwave amplitude A_{MW} to keep T_{π} = 50 ns. The baseband signal for I/Q modulation is generated by another Sony/Tektronix AWG520 which is synchronized with the one used for the gate voltage pulse generation. The microwave signal is single side-band modulated from the local oscillator frequency by applying 80 MHz cosine/sine waveforms to the I/Q ports of the vector signal generator. The unwanted spurious signals are suppressed by applying proper frequency dependent corrections for DC offset, quadrature amplitude and phase. The instrumental noise is much smaller than the value required to reproduce the measured qubit fidelity.

Rapid measurement of the charge state is performed by rf-reflectometry of a sensor quantum dot coupled to an impedance matched resonant circuit which consists of a commercial 1.2 μH SMD inductor and a parasitic capacitance. The resonant circuit operates at its resonance frequency of 206.7 MHz. The reflected signal is amplified by a cryogenic amplifier (Caltech CITLF1) mounted at the 4 K stage of the dilution refrigerator and further amplified and demodulated by room temperature electronics. After the filtering (cutoff frequency of 300 (30) kHz for the left (right) dot), the signal is digitized using an Alazartech ATS9440 digitizer at a sampling rate of 5 MS/s.

Figure S2 shows a typical charge sensor response for the single-shot spin detection. When a spin up state is measured, the charge sensor signal first increases as it tunnels out to reservoir before decreasing to its initial value when a down spin tunnels back into the dot.

To obtain the up spin probability $P_{\uparrow}^{\mathrm{R(L)}}$, the measurement sequence is repeated for 250 to 1000 times per one data point.

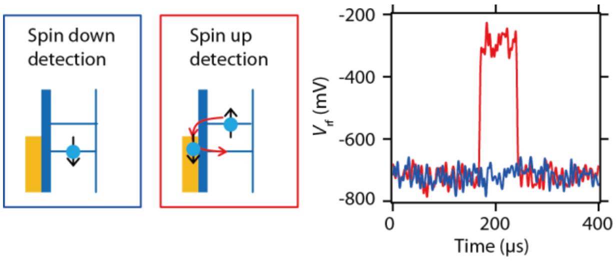

fig. S2. Single-shot spin readout using energy selective readout technique. The pulse sequence in Fig. 2A without the microwave burst is used for this measurement. The blue trace corresponds to the case of spin-down state readout and the red trace corresponds to the spin-up state. When a spin-up state is measured, the charge sensor signal first increases as the electron tunnels out to reservoir before decreasing to its initial value when another spin-down electron tunnels back into the dot.

section S3. Right dot measurement data

Figure S3A shows the measurement result of Rabi oscillations for the right quantum dot. Since the right dot is far from the charge sensor quantum dot placed at the left side of the device, the sensitivity of the sensor is smaller than for the left dot. This causes the reduced readout visibility since a reduced measurement bandwidth (30 kHz) has to be used to keep large enough signal to noise ratio. In this case, some of the fast tunneling events are not measured. The visibility could be enhanced by retuning the gate voltages to reduce the right dot tunnel rate slower than the measurement bandwidth.

Figure S3B shows the Ramsey measurement data. The fit shows a Gaussian decay curve with an exponent of 2. The measured T_2^* of 2.2 μs is comparable to that of the left dot electron spin.

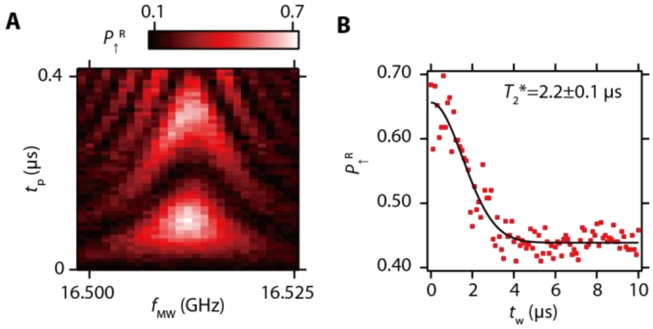

fig. S3. Rabi oscillation and Ramsey measurements of the right quantum dot. (**A**) Measurement result of Rabi oscillation of the right dot spin state measured at B_{ext} = 0.512 T. (**B**) Ramsey measurement result of the right dot spin qubit. The red points show the measurement data and the black solid line shows a Gaussian fitting curve. The obtained 2.2 μs phase coherence time is comparable to that (1.84 μs) in the left quantum dot.

section S4. Discussions on the microwave power dependence

The procedure we use for fitting the Rabi oscillation data in Fig. 3A is as follows. Although the standard nuclear spin dephasing causes non-exponential decay function (Ref. 3), here we empirically use an exponential decay function to fit the data. The source of the exponential decay is unknown in the present work, however, it may be revealed by further detailed observations of the driven qubit noise, for example using the method as described in Ref. 29.

1. We fit the raw data with exponentially decaying function to obtain initial guesses of T_2^{Rabi} and f_{Rabi}

$$P_\uparrow(t_\mathrm{p}) = A\exp(-\frac{t_\mathrm{p}}{T_2^{\mathrm{Rabi}}})\sin(2\pi f_{\mathrm{Rabi}} t_\mathrm{p} + \phi) + B$$

2. We apply a finite impulse response (FIR) high-pass filter with a cutoff frequency of $f_{\mathrm{Rabi}}/2$ and a passband frequency of f_{Rabi} to the raw experimental data for $A_{\mathrm{MW}} \geq$ 0.3 in order to remove the gradual background change.

3. We fit the FIR filtered data with an exponential function to obtain experimental T_2^{Rabi} and f_{Rabi}.

The use of the FIR filter is essential to obtain good fitting curves. Note that

it does not change the damped oscillation term in the unfiltered raw data since the spectrum of the damped oscillations is centered at $f = f_{\mathrm{Rabi}}$ and has a typical width of $1/T_2^{\mathrm{Rabi}} < f_{\mathrm{Rabi}}$. The gradual background change is presumably due to the small electron temperature increase during the readout stage.

From the fitting of the data in Fig. 3C, we notice the empirical dependence of $T_2^{\mathrm{Rabi}} \propto (A_{\mathrm{MW}})^{-4}$ (fig. S4B). If the system temperature T is determined by the microwave heating, the temperature should be proportional to the microwave power $(A_{\mathrm{MW}})^2$. Then the relation $T_2^{\mathrm{Rabi}} \propto 1/T^2$ between the Rabi decay time and the temperature can be obtained. This is consistent with the observation by Dial *et al.* (23) where the relation $T_2^{\mathrm{echo}} \propto 1/T^2$ is observed for a singlet-triplet qubit.

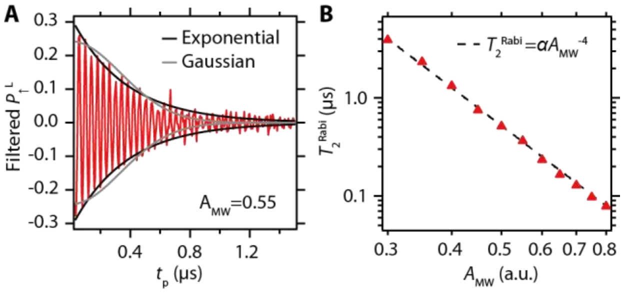

fig. S4. Fitting of Rabi oscillation data. (**A**) Fitting of the damped oscillation at A_{MW} = 0.55. The black solid line shows an exponential envelope $\pm A\exp(-t_p / T_2^{\mathrm{Rabi}})$ and the gray solid line shows a Gaussian envelope $\pm A\exp(-(t_p / T_2^{\mathrm{Rabi}})^2)$. (**B**) Microwave amplitude dependence of the Rabi oscillation decay time. The red triangles show the data and the black dotted line shows fitting with a function $T_2^{\mathrm{Rabi}} = \alpha(A_{\mathrm{MW}})^{-4}$.

As mentioned in the main text, the photon-assisted tunneling can be another dephasing mechanism. In a double quantum dot connected to reservoirs, there are two types of photon-assisted processes. One is the inter-dot photon-assisted tunneling. Since this tunneling process occurs only when the inter-dot energy detuning is the microwave frequency, this process can easily be ruled out by the outcome of the spectroscopy measurement (Fig. 1D).

Indeed, such a strong frequency dependent signal is not observed in the measurement data.

The other photon-assisted tunneling causes the electron exchange between the dot and the reservoir. To exclude this possibility, the measurement to confirm the effect of the coupling to the reservoir is performed (fig. S5). The Rabi decay time measurement is performed for two different operation points with two different Coulomb blockade depths (about 400 or 200 μeV away from the (1,1)-(0,1) charge transition). Due to the slight quantum dot displacement due to the operation point change, the center resonance frequency of EDSR shifts accordingly. Since the microwave transmission is slightly different for the two different center resonance frequencies, T_2^{Rabi} is plotted as a function of f_{Rabi} to account for this change. The measurement result shows basically the same characteristics for the two different operation points, therefore it is unlikely the coupling to the reservoir causes the Rabi decay time decrease measured in this work.

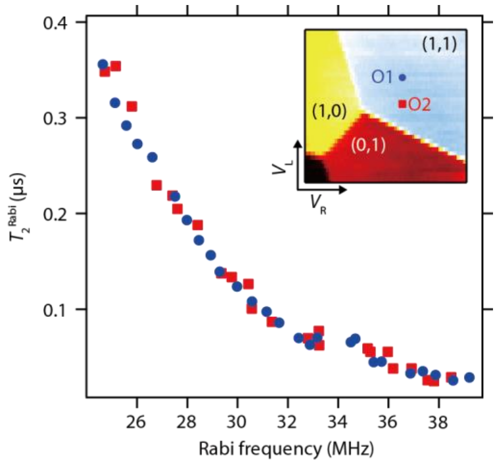

fig. S5. Rabi decay measurement for two different operation points. The operation point 1 (2) (O1 (O2) in the inset charge stability diagram) is about 400(200) μeV away from the (1,1)-(0,1) charge transition line. The two data sets basically showing almost the same characteristic, therefore the effect of the coupling to the reservoir is small for the enhanced dephasing at high microwave amplitudes.

section S5. Randomized benchmarking

Each data point in Fig. 4 is obtained from 12,800 single-shot measurements (800 points per one sequence). The last recovery Clifford gate ensures that the ideal final state to be an eigenstate of σ_z which is up or down spin state. In this measurement, the gates I, ±X, ±Y, ±X/2 and ±Y/2 are used as primitive Clifford gates since these can be implemented by single-step Gaussian microwave bursts. The decomposition of the total 24 single-qubit Clifford gates by these primitive gates results in the average number of

primitive gates per one decomposed Clifford gate of 1.875 (30, 31). The single Clifford gate fidelity F_c^{single} refers to the average gate fidelity per one primitive Clifford gate while the Clifford gate fidelity F_c refers to the average gate fidelity per one decomposed Clifford gate. From the measurement data of the reference sequence, the exponential decay curve $F(m) = A(p_c)^m$ and the Clifford gate fidelity $F_c = (1+p_c)/2$ can be obtained. Then the single Clifford gate fidelity F_c^{single} is calculated using the following equation

$$F_c^{\text{single}} = \frac{1+p_c^{\text{single}}}{2} \sim 1 - \frac{1-F_c}{1.875}$$

where $p_c^{\text{single}} = (p_c)^{\frac{1}{1.875}}$ represents the decrease of the sequence fidelity per single primitive Clifford gate.

To characterize each of the primitive Clifford gate fidelities, the interleaved randomized benchmarking (25) is used. In this measurement, similar exponential decay curves $F(m) = A(p_{\text{gate}})^m$ are obtained and the fidelities for each interleaved gate F_{gate} are calculated from the following equation

$$F_{\text{gate}} = \frac{1+(p_{\text{gate}}/p_c)}{2}$$

where the effect of the randomizing sequence is taken into account by the factor of $1/p_c$.
